# Effect of Parecoxib on Postoperative Pain Management After Total Knee/Hip Arthroplasty: A Systematic Review and Meta-Analysis

**DOI:** 10.7759/cureus.32339

**Published:** 2022-12-09

**Authors:** Sri Nikhil Zallipalli, Rakesh Reddy Bethi, Madhuri Kandru, Nikith Kashyap Dendukuri, Geethanjali Gandla, Sree Sai Siddhardha Vemuri, Harish Peri, Saichand Linga

**Affiliations:** 1 Department of Surgery, Star Hospitals, Hyderabad, IND; 2 Department of Surgery, Bharati Vidyapeeth University Medical College & Hospital, Sangli, IND; 3 Department of Surgery, Coventry University, Coventry, GBR; 4 Healthcare Management, Anglia Ruskin University, Chelmsford, GBR; 5 Department of Surgery, Max Super Specialty Hospital, New Delhi, IND; 6 Department of Surgery, Armed Forces Medical College, Pune, IND; 7 Department of Surgery, South Tyneside and Sunderland NHS (National Health Service) Foundation Trust, Sunderland, GBR

**Keywords:** jadad score, total hip arthroplasty, total knee arthroplasty, vas scores, adverse events, placebo, postoperative pain, parecoxib

## Abstract

Background: Total knee arthroplasty (TKA) or total hip arthroplasty (THA) are frequent procedures used to relieve the symptoms of hip or knee joint dysfunction, enhance disease recovery, and boost patients' quality of life. Nevertheless, postoperative pain has been a significant disadvantage since it strongly impacts patients' postoperative recovery. Parecoxib has been demonstrated to be useful in the management of postoperative pain in a variety of surgical procedures. While parecoxib can help with postoperative pain, its analgesic and unfavourable effects in TKA/THA patients have not been well studied.

Methods: A systematic search of peer-reviewed articles was conducted through the PubMed database, Google Scholar, and Cochrane library to retrieve related studies published in the English language that met inclusion and exclusion criteria. The publication date was restricted to the past 10 years (2012-2022). Results were analyzed using Review Manager software (RevMan version 5.4.1, The Cochrane Collaboration, 2020). The quality of the studies included was assessed using Jadad scores. Risk ratios (RR) standard mean difference (SMD) and 95% confidence intervals (CI) were calculated to analyze the primary and secondary endpoints.

Results: Eleven randomized controlled trials covering 1911 patients who underwent TKA/THA were selected. The pooled results indicated that the parecoxib group has lower visual analogue scale (VAS) scores than the placebo group. However, there was no significant difference in the secondary endpoint. The Jadad scores ranged from 3 to 5 and most of the studies were of high quality.

Conclusion: The results of our meta-analysis indicate that parecoxib has a better analgesic effect compared to placebo. It alleviates postoperative orthopaedic pain without raising the risk of adverse events.

## Introduction and background

Orthopedic surgery is one of the most prevalent medical procedures, and its prevalence has increased over the last decade. On the other hand, they are considered the most painful, with moderate-to-severe postoperative discomfort relatively common [1]. In the initial phases following total knee arthroplasty (TKA) and total hip arthroplasty, pain management is critical since rapid and thorough physical rehabilitation and exercise are recommended but may be limited by discomfort [2]. Moreover, there is a high likelihood of poor compliance following surgery, with pain being the most important determinant. Thus, following major orthopedic surgeries, the choice and usage of appropriate analgesics are critical aspects to guarantee that the patient can relax at night and endure treatment during the daytime [3].

Although opioid administration is indicated as the first-line treatment for postoperative pain, it is linked with a variety of side effects such as fatigue, disorientation, nausea, itchiness, and digestive problems. These side effects upset the patient and may necessitate additional therapies to address them, thereby prolonging mobility and extending recovery/hospitalization durations [4]. A comprehensive strategy for postoperative analgesia provides benefits over using opioids alone. Because of the elevated incidence of perioperative bleeding, nonselective nonsteroidal anti-inflammatory medications (NSAIDs) are not advised as first-line supplementary analgesics in the perioperative context [5]. Conversely, selective cyclooxygenase-2 (COX-2) inhibitors (coxibs) reduce postoperative discomfort while having little effect on platelet activity or intraoperative loss of blood. As a result, in combination with morphine, coxibs could deliver adequate additional analgesia [6].

Parecoxib is a COX-2 selective inhibitor that can be given intravenously or intramuscularly for the quick treatment of postoperative pain. It is a prodrug that is promptly metabolized in the body to create the active metabolite, valdecoxib [7]. Parecoxib has been shown in clinical studies to be beneficial in managing postoperative pain following oral surgery [8], orthopedic surgery [9], and abdominal surgery [10]. Additional research has found no substantial effects on platelet activation or the gastrointestinal mucosa. As a basis, parecoxib has been licensed for the management of postoperative pain in a number of nations. Coxibs appear to be a good therapy for immediate postoperative pain, according to systematic studies [11]. Parecoxib has been demonstrated to be useful in the management of postoperative pain in a variety of surgical procedures. Nevertheless, it is ambiguous if the advantage of parecoxib in other procedures extends to individuals having TKA/THA. There have been few studies that look at the analgesic effectiveness and opioid-sparing benefits of postoperative parecoxib therapy in patients following complex orthopedic surgeries [12]. 

The current systematic review and meta-analysis were performed to evaluate the effect of parecoxib on postoperative pain management after TKA/THA.

## Review

Methods

Search Strategy

This review has been carried out following the Preferred Reporting Items for Systematic Reviews and Meta-Analyses (PRISMA) 2020 guidelines [13]. A comprehensive literature search was conducted on online electronic databases PubMed, Google Scholar, and Cochrane to retrieve studies that are relevant to the review. In addition, the search was performed in a relevant bibliographic database from the University of Chester. The initial search was carried out in January 2022 and was completed with a new search to update the review in April 2022. The following combinations of keywords were used: “parecoxib,” “orthopedic surgery,” “total hip/knee arthroplasty,” “pain,” “analgesia,” “randomized controlled trial,” and other related MeSH terms. The search limits were articles published between 2012 and 2022. A PRISMA flow diagram was used to summarize the key processes involved in the search phase. Following the framework of the chosen keywords, the complete collection of records was analyzed to identify potential duplication of publications collected from various sources; the remaining papers were then evaluated in full text for eligibility to identify all those meeting the inclusion criteria.

Applied search in PubMed: (((((total hip arthroplasty) OR (total knee arthroplasty)) OR (orthopedic surgery)) OR (knee replacement)) AND (parecoxib)) AND (randomized)

Applied search in Cochrane library: (parecoxib):ab AND ("total knee arthroplasty"):ab AND (pain):ab (Word variations have been searched)

Data Extraction

The titles and abstracts of all records acquired utilizing the database search method were independently reviewed by two authors (SNZ and RRB). If additional data was needed to assess eligibility or if there was disagreement among authors, the full text was evaluated. Language restriction was applied and only English language articles were considered. Disagreements among authors were first sorted by discussion and resolution with the third author. The major information retrieved comprised the first author's name, year of publication, test type, sample size, intervention, kind of procedure, and outcomes assessed in each research.

Eligibility Criteria

Eligibility criteria were based on the Population, Intervention, Comparison, Outcomes, and Study (PICOS) framework.

Population: Patients > 18 years of age who underwent TKA/THA surgery were considered.

Intervention: 40 mg parecoxib was used in the experimental group and placebo in the control group.

Comparison: A comparison group was necessary for all studies (of the same condition). An alternate treatment group could not be used as the comparison group.

Outcomes: We evaluated the results using the following specified interpretations: (i) pain score was assessed using a visual analog scale (VAS); (ii) adverse events were documented independently of therapy or probable causal link with parecoxib.

Study design: Only randomized controlled trials (RCTs) were taken into account. Publications that described the viability and fit-out of therapies were not accepted. Data extraction for all qualifying trials was conducted separately by three authors (SNZ, RRB, and NKD), and was cross-checked for integrity by a fourth author (GG). Whenever evidence was deemed to be lacking for either the methodological evaluation or data retrieval processes, the principal authors of eligible studies were approached.

Exclusion Criteria

Duplicated studies, animal experiments, non-RCTs, reviews, and case reports; studies with incomplete data; studies that did not focus on TKA/THA.

Assessment of Study Quality and Risk of Bias

The quality of the RCTs included in the meta-analysis was independently assessed by the authors using the Cochrane Handbook [14]. The following criteria were used to determine the Jadad rating: (i) was the research a randomized trial; (ii) was the randomization method explained and applicable; (iii) was the study mentioned as double-blinded; (iv) was the technique of double-blinding acceptable, and (v) was there a summary of dropouts and withdrawals. Using Jadad ratings, each author appraised the quality of the trials (low= 0-1; medium= 2-3; high= 4-5).

Statistical Analysis

Data were analyzed using Review Manager software (RevMan version 5.4, The Cochrane Collaboration, 2020). The selected papers were pooled and weighted. In either a random-effects or a fixed-effects model, the relative risk (RR) and 95% confidence interval (CI) were determined. The χ2 test was used to examine heterogeneity, and the I2 statistic was used to quantify it. If there was heterogeneity (P0.01 or I2>50%) across the trials, a random-effects model was chosen; otherwise, a fixed-effects model was used. For the meta-analyses, a random-effect model was utilized.

Results

Study Characteristics

**Figure 1:**
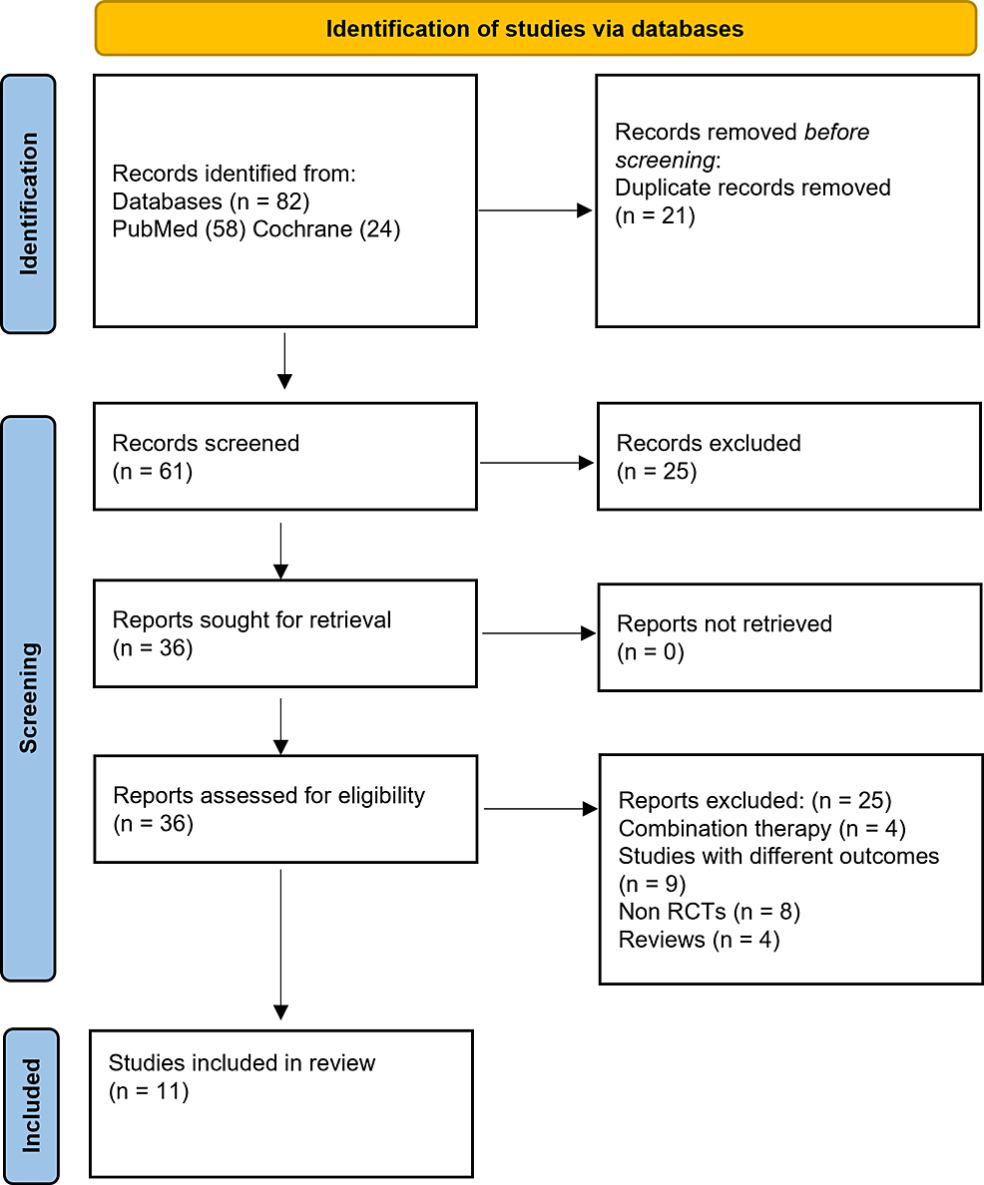
PRISMA flow diagram illustrating the search strategy and selection of relevant articles for the qualitative analysis PRISMA: Preferred Reporting Items for Systematic Reviews and Meta-Analyses; RCT: randomized control trial

According to the PRISMA recommendations, 82 relevant studies were retrieved. The titles and abstracts of the articles were reviewed to exclude publications that were not relevant. We rejected studies that did not fulfill the given criteria after reviewing the complete text of those that qualified. Finally, as shown in Figure 1, 11 articles [4,9,15-23] representing 1911 patients were included in our meta-analysis. Of the 1911 patients, 940 (49.2%) were assigned to the parecoxib group, whereas 971 (50.8%) were assigned to the placebo group (Table 1). This meta-included analysis's studies were all RCTs.

**Table 1:** Baseline characteristics of the included studies TKA: total knee arthroplasty; THA: total hip arthroplasty; T: treatment group; C: control group; IV: intravenous; VAS: visual analog score; AEs: adverse effects

Author and year	Procedure	Site	Timing	Sample size (T/C)	Intervention (T/C)	Outcome
Bian et al., 2018 [9]	unilateral TKA	single-centered	Pre, Post	88 (46/42)	Parecoxib 40 mg IV Q12h; Placebo	VAS scores, AEs
Peng et al., 2018 [20]	unilateral THA	two-centered	Pre	94 (48/46)	Parecoxib 40 mg IV once; Placebo	VAS scores, AEs
Ma et al., 2021 [22]	bilateral TKA	single-centered	Pre, Post	56 (28/28)	Parecoxib 40 mg IV Q12h; Morphine	Pain scores, AEs
Laoruengthana et al., 2020 [21]	unilateral TKA.	single-centered	Pre, Post	100 (50/50)	Parecoxib 40 mg IV Q12h; 48h Ketorolac 30mg IV Q12h; 48h	Pain scores
Xiao et al., 2019 [23]	unilateral THA	single-centered	Pre, Post	141 (69/72)	Parecoxib 40 mg IV Q12h; Placebo	VAS scores, AEs
Essex et al., 2018 [19]	unilateral TKA	multi-centered	Post	116 (58/58)	Parecoxib 40 mg IV once; Placebo	AEs
Diaz-Borjon et al., 2017 [4]	TKA/THA	multi-centered	Post	281 (142/139)	Parecoxib 40 mg IV; Placebo	AEs
Mu et al., 2017 [18]	TKA/THA	two- centered	Post	620 (310/310)	Parecoxib 40 mg IV Q12h; Placebo	AEs
Camu et al., 2017 [17]	THA	multi-centered	Post	181 (72/71/38)	Parecoxib 40 mg Propacetamol 2g; Placebo	Pain scores, AEs
Zhu et al., 2014 [16]	TKA	single-centered	Pre, Post	134 (67/67)	Parecoxib 40 mg IV Q12h; Placebo	VAS scores
Zhu et al., 2016 [15]	TKA	single-centered	Post	100 (50/50)	Parecoxib 40 mg IV Q12h; Placebo	VAS scores

Assessment of Risk of Bias

**Figure 2:**
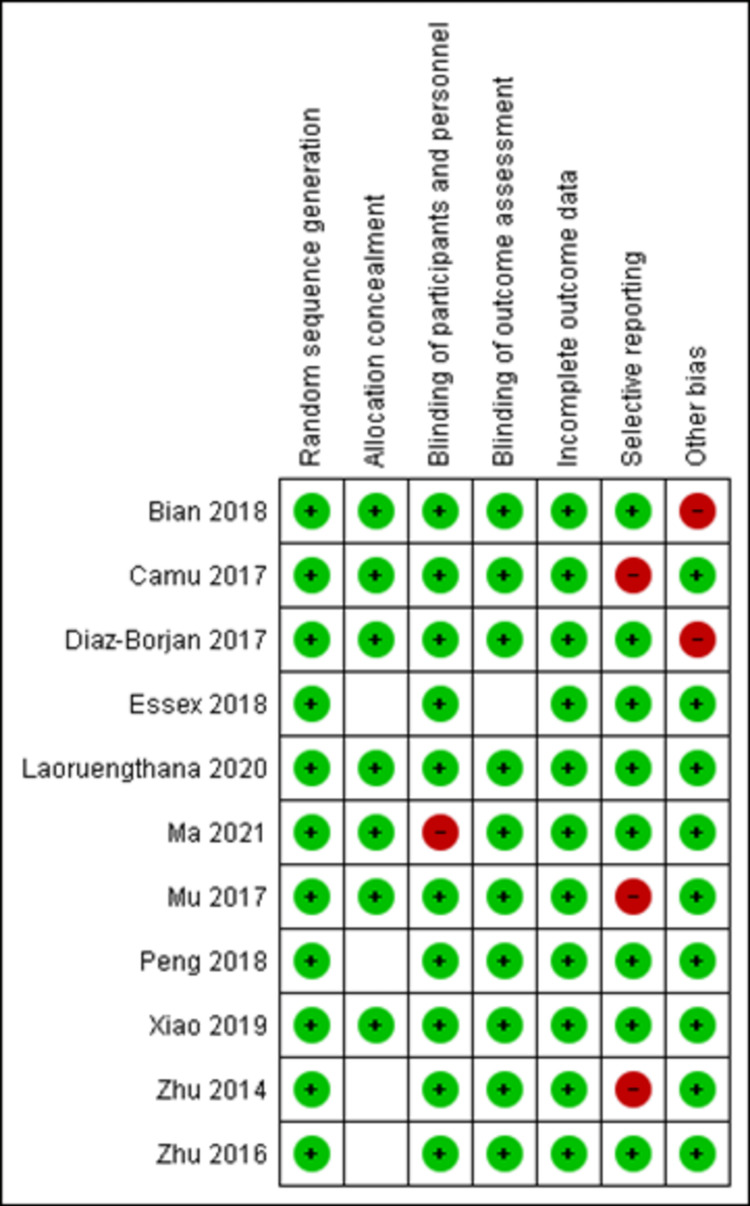
Risk-of-bias summary Review of authors' judgement about each risk-of-bias item for each included studies [4,9,15-23]

**Figure 3:**
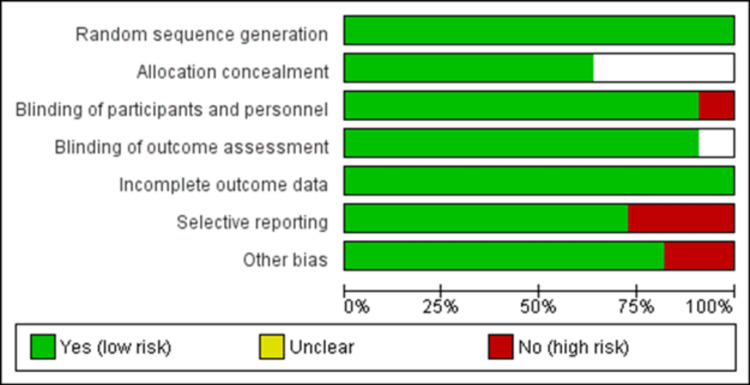
Risk-of-bias graph Review of authors' judgement about each risk-of-bias item for each included comparison

RevMan version 5.4 was used to assess the methodological quality of the included studies. The scale comprises the following definitions: (i) randomization (selection bias); (ii) allocation concealment (selection bias); (iii) double blinding (performance bias); (iv) outcome assessment blinding (detection bias); (v) incomplete outcome data (attrition bias); (vi) selective reporting (reporting bias), and (vii) other biases (possible unknown bias). The studies were divided into three categories: low risk of bias, unclear, and high risk of bias. Included studies were evaluated and it was found that randomization was reported in all studies evading possible selection bias. Allocation concealment was unclear in four studies [15,16,19,20]. In one study, only assessors were blinded leading to performance bias [19]. Selective reporting was observed in three studies leading to reporting bias [16-18]. The data are illustrated in Figures 2, 3.

Assessment of Methodological Quality

The quality assessment of each study was analyzed using Jadad scores and is described in Table 2. The study quality checklist items that were scored ranged from 0-5 points out of a total of 5 points. Studies were classified into three levels as follows: low quality (0-2), medium quality (3-4), and high quality (5). Of 11 studies included, seven studies were of high quality with a score of five points each; two studies [15,19] scored four points each and two articles [4,22] scored three points each indicating medium quality. Overall, the studies included in the analysis were of high quality with reliable data.

**Table 2:** Jadad scores given by the authors for the studies included in the analysis The table demonstrates the quality of the included studies based on a 5-point scale with randomization and allocation concealment as scaling elements

Study	Randomized	Double binding	Described appropriately?		Withdrawal and dropouts	Total score Out of 5
			Randomization	Double blinding		
Bian et al., 2018 [9]	1	1	1	1	1	5
Camu et al., 2017 [17]	1	1	1	1	1	5
Diaz-borjan et al., 2017 [4]	1	1	0	0	1	3
Essex et al., 2018 [19]	1	1	1	0	1	4
Laoruengthana et al., 2020 [21]	1	1	1	1	1	5
Ma et al., 2021 [22]	1	0	1	0	1	3
Mu et al., 2017 [18]	1	1	1	1	1	5
Peng et al., 2018 [20]	1	1	1	1	1	5
Xiao et al., 2019 [23]	1	1	1	1	1	5
Zhu et al., 2014 [16]	1	1	1	1	1	5
Zhu et al., 2016 [15]	1	1	1	0	1	4

Primary endpoints

VAS Scores at Rest

**Figure 4:**
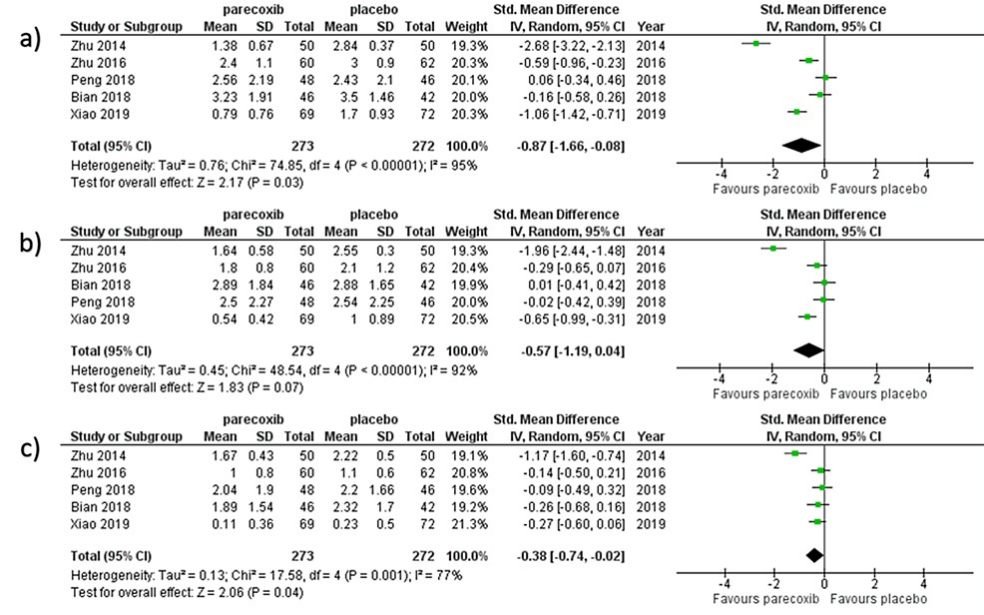
Forest plot of VAS scores at rest obtained at various timelines: 12 hours, 24 hours, and 48 hours After excluding the articles with inadequate data, five studies were pooled to analyze the VAS scores [9,15,16,20,23]

We selected the random-effect model to perform the meta-analysis since there were significant heterogeneities. Five studies reported VAS scores at rest within two days after surgery [9,15,16,20,23]. VAS scores at rest were recorded for 545 patients in total, with 273 allocated to the parecoxib group and 272 allocated to the placebo group. As shown in Figure 4, VAS scores throughout rest in the parecoxib group were considerably lower than those in the placebo group at 12 hours, 24 hours, and 48 hours (SMD − 0.87, 95%CI − 1.66 to − 0.08, I2 = 95%; SMD − 0.57, 95%CI − 1.19 to − 0.04, I2 = 92%; SMD − 0.38, 95%CI − 0.74 to − 0.02, I2 = 77%, respectively). To estimate the degree of heterogeneity among the publications, the random effect model was used.

VAS Scores at Movement

**Figure 5:**
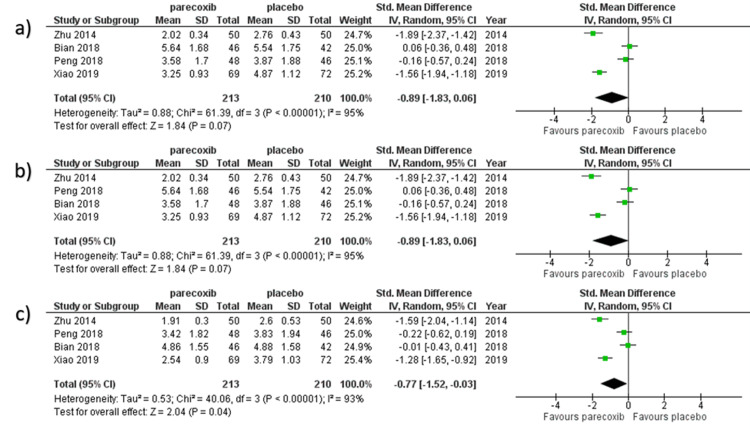
Forest plot of VAS scores at movement obtained at various timelines: a) 12 hours, b) 24 hours, and c) 48 hours. The studies with data on experimental group and placebo group VAS scores recorded at moment were pooled [9,16,20,23].

We selected the random-effect model to perform the meta-analysis since there were significant heterogeneities. Four studies reported VAS scores for movement within two days after surgery [9, 16, 20, 23]. In all, 423 patients' VAS scores following movement were reported, with 213 allocated to the parecoxib group and 210 allocated to the placebo group. At 12 hours, 24 hours, and 48 hours, VAS scores following movement in the parecoxib group were considerably lower than those in the placebo group (SMD − 0.89, 95%CI − 1.83 to − 0.06, I2 = 95%; SMD − 0.89, 95%CI − 1.83 to − 0.06, I2 = 95%; SMD − 0.77, 95%CI − 1.52 to − 0.03, I2 = 93%) respectively as shown in Figure 5. To estimate the degree of heterogeneity among the publications, the random effect model was used.

Secondary endpoints

**Figure 6:**
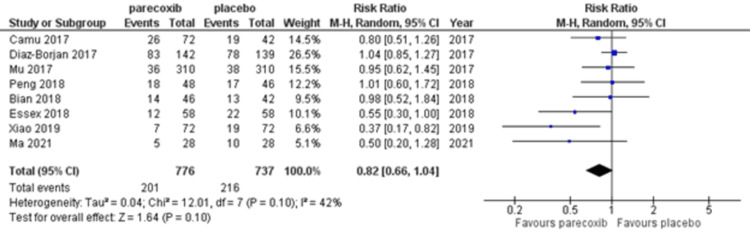
Forest plot of adverse events risk ratios in parecoxib and placebo groups of the included studies The included studies for secondary endpoints: [4,9,17,18,20,22,23]

Pooled data from eight RCTs [4,9,17-20,22,23] with 776 and 737 patients in the parecoxib and placebo groups, respectively, revealed no significant difference in the occurrence of adverse events (RR 0.82, 95% CI 0.66-1.04) as shown in Figure 6. All of the events, such as nausea, headache, and constipation were present; however, other adverse effects were minimal as shown in Table 3. There were no gastrointestinal hemorrhage problems.

**Table 3:** The total number of adverse events occurred in experimental and control groups with individual adverse event type According to the table data, there was no significant difference between parecoxib and control groups

Sub group	Parecoxib	Placebo
Cardiovascular	12	9
Constipation	2	2
Fatigue	4	0
Fever	7	18
Headache, dizziness	22	19
Nausea, vomiting	72	84
Neurological	1	2
Others (Anemia, wound infection, sepsis)	18	33
Pruritis	20	10
Pulmonary	4	9
Rashes	7	5
Sleep disturbances	12	6
Urine retention	1	3
Venous thrombosis	19	16
Total events	201	216

Discussion

Following orthopedic procedures such as TKA/THA, pain management is strongly connected to quick recovery, effective rehabilitation, and patient satisfaction. In this analysis of TKA/THA patients, postoperative administration of parecoxib resulted in significantly improved pain ratings compared to placebo at 12, 24, and 48 hours. The current meta-analysis was carried out to assess the effect of parecoxib on postoperative pain in patients undergoing TKA/THA. The findings of the meta-analysis showed that parecoxib outperformed the placebo with a statistically significant difference. The VAS ratings were lower after 12, 24, and 48 hours of the first dosage of 40 mg parecoxib IV compared to the placebo group, showing that parecoxib had a strong analgesic effect. Unlike earlier studies that looked at orthopedic procedures as a whole [24], our meta-analysis focused just on TKA/THA. According to our knowledge, this is also the first meta-analysis to investigate the role of parecoxib in postoperative pain control following TKA/THA. The primary outcomes reveal that patients taking parecoxib had significantly lower VAS ratings at rest or during movement (within 48 hours) than placebo participants. Analyses of safety outcomes suggest that patients on parecoxib experience fewer adverse events than placebo individuals, although there was no significant difference.

According to a pooled study of 28 RCTs, parecoxib is distributed in several regions in the kidney and regulates a range of renal functions, such as salt and water retention/elimination and systemic osmoregulation. As a result, there is a possibility of renal dysfunction or damage when using parecoxib. In a study of summarized clinical trial data, researchers discovered that the prevalence of renal dysfunction and damage was modest and consistent across the parecoxib (1.0%) and placebo (0.9%) arms [25]. Furthermore, the glomerular and tubular functionalities were temporarily altered in all patients following orthopedic surgery; nonetheless, the variations between the parecoxib group and placebo group were minor and clinically insignificant [9]. Patients with a creatinine clearance of 30 mL/min or who are susceptible to fluid retention are also at the possibility of deteriorating kidney performance; thus, parecoxib should be started at the smallest indicated dose and the patient's renal capability should be regularly checked [26].

In the included trials, 40 mg of parecoxib was given intravenously twice a day for three days following surgery. Earlier studies have shown that this dosing strategy enhances postoperative pain control by improving analgesic efficacy and decreasing opioid demand [27]. Although parecoxib offers an analgesic effect when provided before or after surgery, few trials have evaluated the time of parecoxib administration, and the findings are equivocal. In studies of general surgery and THA, preoperative administration of parecoxib offered higher pain alleviation than postoperative treatment [28]. Nevertheless, there was no significant change in pain severity comparing preoperative and postoperative administration of parecoxib in another study on THA. Additional research is needed to evaluate whether preoperative parecoxib therapy benefits postoperative therapy in patients undergoing orthopedic surgeries.

Important limitations impacting the present meta-analysis: (i) Numerous basic parameters were not addressed (older age or co-morbidities), which may have introduced mixed bias; (ii) Since our meta-analysis is based on a few RCTs, we accept that employing a small number of trials increases the likelihood of selection uncertainty; (iii) The end variables documented in the selected studies were utilized. Consequently, determining the impact of the basic parameters on the outcomes of this meta-analysis is challenging;; (iv) Due to the limitations of the included trials, we did not investigate interactions between subgroups; and (v) The findings of the primary endpoints were very diverse, and sensitivity analysis was unable to establish the root cause of heterogeneity.

## Conclusions

While there were inherent constraints to this meta-analysis, the data shows that parecoxib is beneficial and considered a convenient alternative for postoperative pain treatment in TKA/THA patients. However, the data analysis of the adverse events in the secondary endpoint suggests that there was no significant difference between the experimental group and the control group. Considering the inclusion of few studies in the current meta-analysis, the findings were a representation rather than a conclusion of the parecoxib effect. To assess the benefits of parecoxib for postoperative pain, additional high-quality RCTs are needed. Nevertheless, parecoxib is a safe and effective drug in perioperative and postoperative pain management in TKA/THA.
